# The control of hookworm infection in China

**DOI:** 10.1186/1756-3305-2-44

**Published:** 2009-09-24

**Authors:** Qi Zheng, Ying Chen, Hao-Bing Zhang, Jia-Xu Chen, Xiao-Nong Zhou

**Affiliations:** 1 National Institute of Parasitic Diseases, Chinese Center for Disease Control and Prevention, Shanghai 200025, PR China

## Abstract

**Background:**

Hookworm is still one of the three main soil-transmitted helminths prevalent in China, and 39 million cases infected with hookworm were estimated in China in 2006.

**Results:**

The main approach to the control of hookworm infections in China consists of large-scale deworming, rebuilding sanitation systems in rural areas and health education. The availability of low-cost, safe and single-dose albendazole make large-scale deworming programs possible in China. Currently, sanitary latrines with three-cells are recommended by government for the control of soil-transmitted helminths, since 35% of helminth infections and 83% of worm eggs could be reduced after using this kind of sanitary latrine. In addition, economic prosperity contributes greatly to the reduction of hookworm prevalence, but the inequity of economic and social development among different regions of China provides a scenario that the worst threat of hookworm infection is located in the poorest areas of southern and central China. Therefore, it is necessary to put more investments into prophylaxis and treatment of hookworm in these poor regions.

**Conclusion:**

Although the prevalence of hookworm infection has fallen significantly in the last 15 years in China, the current strategy for controlling hookworm infections still needs to be strengthened along with the three-pronged approach, e.g. distributing anthelmintic drugs in schools and undertaking large-scale of hookworm deworming, improving water supplies and sanitation, and proper health education.

## Background

**Figure 1:**
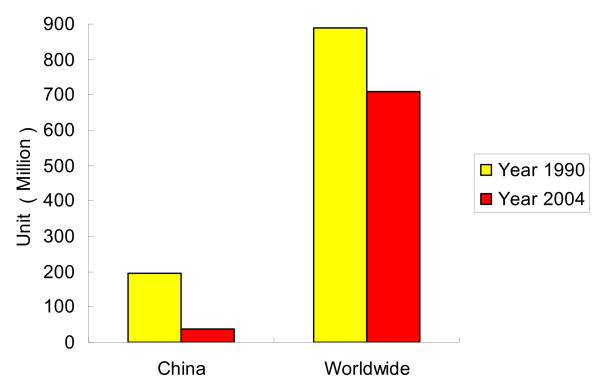
**The number of hookworm infected cases in China and worldwide (unit: million)**.

Hookworm is one of the three main soil-transmitted helminths endemic in China [1]. Hookworm disease is mainly caused by human infection with *Necator americanus *or *Ancylostoma duodenale*, and rarely with *Ancylostoma brazilienese, Uncinaria stenocephala*. Although the prevalence of hookworm has been controlled through effective control strategies in the last ten years, it still remains an important public health problem wherever rural poverty occurs, especially in the tropics and subtropics. In 2006, there were 576 million cases of hookworm disease estimated worldwide and 39 million in China [2,3]. Compared with the data reported in 1990, the prevalence of hookworm had fallen much more rapidly in China (reduction rate: 63.7%) than worldwide (reduction rate: 35.2%) in 2004 (Figure 1) [4,5].

Hookworm disease is one of the most important causes of physical growth retardation and intellectual development retardation in humans [6]. In spite of its educational, economic, and public-health importance, it remains largely neglected by the medical and international community [7]. This neglect stems from three features: first, poverty: the people most affected are the world's most impoverished, particularly those who live on less than US$2 per day. For instance, most of the high infection areas are in the underdeveloped regions in China, such as Guangxi province and Yunnan province (Table 1). Second, chronic illness: hookworm infection causes chronic illness and has insidious clinical symptoms and signs, which are not only neglected by infected cases themselves, but also by medical doctors [8]. Third, difficulty in evaluation: quantification of the effects of hookworm infections, such as the burden of disease and its influence by economic development and education level, are difficult to estimate [9]. Since the early 1990s, however, the Chinese government has begun to recognize the importance of hookworm infections and made a series of plans to reduce hookworm infections [10]. Therefore, this paper focuses on describing the control efforts, in addition to considering the transmission patterns and factors that influence hookworm infections in China.

**Table 1:** The geographic distribution of hookworm infections in China

**Pathogens**	**Morbidity**	**Geographic distribution**
*Necator americanus*	Hookworm disease	Chongqing, Guangxi, Fujian
*Ancylostoma duodenale*		Sichuan, Hainan, Guizhou

*Ancylostoma brazilienese*	Cutaneous larva migration	South China and Coastal regions
*Uncinaria stenocephala*	Cutaneous larva migration	South China and Coastal regions
*Ancylostoma caninum*	Eosinophilia enteritis	Coastal regions

## Control of hookworm infections

The Chinese government has devoted much man-power, materials and financial resources to reduce the prevalence and intensity of hookworm infections in the past 15 years [11]. In 2005, the Chinese Ministry of Health issued the "National Control Program on Important Parasitic Diseases from 2006 to 2015" [12], with its target to reduce the prevalence of helminth infections by 70% until 2015. In the program, a three-pronged approach was proposed to reach this target, comprising: (1) large-scale deworming with a benzimidazole, (2) providing clean water and adequate sanitation, and (3) health education programs.

### Large-scale deworming with a benzimidazole

With the launch of the deworming program by the Chinese Ministry of Health in 2005, it is intended to conduct large-scale deworming with effective drugs in areas where the infection rate of soil-transmitted helminths is more than 50%, and with its target population > 3 years of age. Furthermore, selected chemotherapy will be conducted for the school-aged children and farmers in areas where the infection rate of soil-transmitted helminths is more than 10%. For this, two essential issues in the program need to be confirmed. The first question to be solved is how to select the best drug affordable for the target population in the program when mass or selective chemotherapy is performed. Then, how will the efficacy of the program be monitored?

In the past 30 years, four kinds of drugs have been used in the large-scale treatment of hookworm infections in China, namely levamisole, pyrantel pamoate, mebendazole and albendazole [13]. Because of the low cure-rate against hookworm, levamisole is now rarely used against hookworm infections, and pyrantel pamoate is more regarded as an alternative drug [14,15]. So, in order to tackle the multi-infection problem of soil-transmitted helminths that are widespread in China [16,17], mebendazole and albendazole are the drugs most commonly used currently in China [18]. Another reason to use benzimidazoles in the control program is that the drugs' effects on worms can last for several days, because these drugs are able to bind to β-tubulin of the nematodes and therefore inhibit microtuble polymerisation of the parasites [19].

Although both albendazole and mebendazole are considered as broad-spectrum anthelmintic agents, difference in cure-rates and egg reduction rates affect their use in clinical practice. According to a recent meta-analysis of the efficacy of drugs against soil-transmitted helminth infections, the efficacy of single-dose oral albendazole and mebendazole against hookworm infections is 72% and 15%, respectively [20]. Therefore, several doses of mebendazole are commonly needed in the treatment against hookworm infections (Table 2) [21]. Besides, mebendazole is poorly absorbed from the gastrointestinal tract. Hence, its therapeutic activity is largely confined to adult worms. In contrast, albendazole is better absorbed, the drug is then metabolised in the liver to the anthelmitically active albendazole sulphoxide. Two enantiomeric forms of albendazole sulfoxide have been identified in plasma, tissue locations of the parasites and within the target helminths themselves [22]. Furthermore, treatment with a single 400 mg oral dose of albendazole gives cure rates of 46%-100% for hookworm cutaneous larva migrants [23]. Another advantage of the drug is that there are no systemic toxic effects observed at the dosages used to treat soil-transmitted helminth infections since it has been developed [24]. However, some side effects, such as transient abdominal pain, diarrhoea, nausea, dizziness and headache, commonly occur [25]. Because benzimidazole anthelmintic drugs are embryotoxic and teratogenic in pregnant rats [26], there are concerns about their use in children younger than 24 months and during pregnancy [27,28]. Therefore, in China it is recommended not to use benzimidazole in these two populations [29,30].

**Table 2:** The dosages of drugs used in the regular and periodic treatment of hookworm infections in China

**Drugs**	**Dosage**	
	
	**Adults**	**Children**
Albendazole*	400 mg once	400 mg once
Mebendazole*	100 mg twice a day for 3 days	100 mg twice a day for 3 days
Pyrantel pamoate	20 mg/kg (maximum dose 1 g) for 3 days	20 mg/kg (maximum dose 1 g) for 3 days
Levamisole	2·5 mg/kg once; repeat after 7 days in heavy infection	2·5 mg/kg once; repeat after 7 days in heavy infection
Tribendimidine	400 mg once	200 mg once

* based on a recommendation in the Report of the WHO informal consultation on the use of praziquantel during pregnancy and lactation and albendazole/mebendazole in children under 24 months [30].

Many large-scale deworming programs showed that the incidence rate of adverse events of albendazole is less than 5%. In China, albendazole is an OTC (over the counter) drug and it is normally affordable by Chinese people. In China, the retail price for one single dose (400 mg) of albendazole is less than US$0.3, though in the wholesale markets, 400 mg albendazole costs only US$0.02-0.03. Therefore, the availability of low-cost, safe and single-dose albendazole makes large-scale deworming programs possible in China. Particularly in most of rural areas the primary health care system has been implemented, and the treatment cost of anthelmintic drug is almost free for individuals.

In order to ensure progress in the large-scale deworming projects for the control of soil-transmitted helminth infections (including hookworm infection), the Ministry of Health selected 12 counties from 11 provinces in a pilot soil-transmitted helminth control program in 2005. Twice a year albendazole or pyrantel pamoate combined with albendazole was administrated to all residents above 3 years of age [31]. The results from those pilot counties showed that compliance rates reached about 90% and that the infection rate with hookworm fell by 57% after twice community-wide treatment within one year. According to the reported data, after one community-wide treatment, the hookworm infection rate returned to 80% of the pretreatment rate within 30-36 months in regions where further therapy was not provided [32]. In consideration of the fact that the high rates of post-treatment hookworm reinfection and other factors that limit the success of large-scale chemotherapy programs, it is suggested that large-scale periodic treatment should be conducted in those areas where helminth infection is more than 50%. But whether launching such regular mass chemotherapy may encourage drug resistance to develop [33]. Even though resistance has not been clearly demonstrated yet in human hookworm infections, it is most likely to the drug resistance could be introduced if these mass chemotherapy treatment campaigns are sustained [34]. The government should consider this potential problem. The use of alternative or new drugs, such like tribendimidine, is one way to solve this problem[35].

### Sanitation at rural households

One of the objectives issued in the "National Control Program on Important Parasitic Diseases during 2006-2015" was to build hygienic sanitary latrines with a coverage rate at county level above 60% in the rural areas by 2010, and above 80% by 2015. This defined objective was mainly based on the evidence that the transmission of hookworms and other helminths is extremely difficult to eliminate in undeveloped countries with inadequate water and sanitation [36]. Health problems caused by the lack of safe water are exacerbated by poor sanitary conditions [37]. Traditionally, Chinese households collect human waste and transport it to the fields for direct use as fertilizer, often without further treatment. Most people become infected with hookworm larvae when they work on these contaminated soils [38]. Data have shown that in 1997 about 90% of rural households had some sort of household latrine, but most of these facilities were rudimentary, and only provided temporary storage of wastes. About 97.5% latrines do not have any protection function from soil-transmitted helminthes [39].

**Figure 2:**
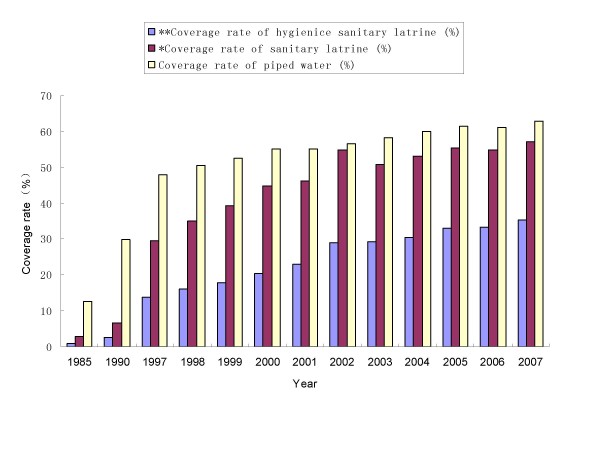
**The coverage rate of hygienic sanitary latrine, sanitary latrine and piped water in rural area of China from 1985 to 2007 (Data of 1997-2007 from the official website of Ministry of Health of People's Republic of China)**.

During the past decades much progress has been achieved in terms of improving the provision of clean water and sanitation. For example, the government has quadrupled investments in the water supply and sanitation during the current Five-Year Plan (2006-2010), in addition to the fact that the coverage rate of piped water and latrines was increased markedly in the past two decades (Figure 2). In particular, the three-cell latrine has been recommended by the government to be built in rural areas. It is a latrine with a three-cell pit, full walls, and a roof in which the pit is airtight and has a cover. Compared with the normal latrines, the three-cell latrine can reduce about 35% morbidity in populations that live in helminth epidemic area by killing 83% of helminth eggs [40,41].

Another example of the promotion of building sanitary latrines is that nine ministries and commissions of the Chinese government united to put forward a program for "health promotion for farmers" with its target that the coverage rate of household methane latrines should be increased by more than 16% by 2010. The household methane latrine is one kind of hygienic sanitary latrine that the government subsidizes in transmission areas where helminths are prevalent. It can reduce the concentration of live helminth eggs after treatment with methane more than other hygienic sanitary latrines. So, with the aid of the government, the coverage rate of hygienic sanitary latrines can increase steadily [42]. The intervention by building household methane latrines will not only prevent soil-transmitted helminths but also have important economic benefits. In one year, a methane latrine of one family with four members, can provide free heat energy that is equal to 400 kg standard coal burns, and also can provide 7-10 tons of clean organic fertilizer. All these benefits sum up to US$120 per year, which encourages people to reconstruct their latrines as methane latrines [43]. Thus, a marked reduction of hookworm infections was observed after the launching this kind of program. In 2002, according to the water and environmental sanitation projects aided by UNICEF, the infection rate of soil-transmitted helminths in Shu Cheng county, Anhui province, was reduced from 28.6% to17.5% after improved water supply projects were implemented in the region [44].

### Health education

Another control strategy of the "National Control Program on Important Parasitic Diseases during 2006 to 2015" is health education, with the goal that awareness of prevention of soil-transmitted helminths should be above 70%, and healthy behavior rate should be above 60% and 80% by the end of 2010 and 2015, respectively. This target was drafted because some behaviors, such as working in the field without shoes or eating uncooked vegetables are directly related to the transmission of hookworm infections [45]. Most of the people who become infected with hookworm do not practice hygienic behavior [46].

In order to reach these targets, local governments made a concerted effort to promote good health-related behaviors. In most rural areas, a network constituted by several non-government organizations, such as National Patriotic Health Campaign Committee, the All-China Women's Federation, the Communist Youth League of China, work with local Centers for Disease Control (CDCs) and schools, in order to lead health education campaigns encouraging a wide range of hygienic behaviors. Its aim was to popularize the knowledge of many basic health behaviors, such as the importance of washing hands with soap before eating and not drinking unboiled water.

**Figure 3:**
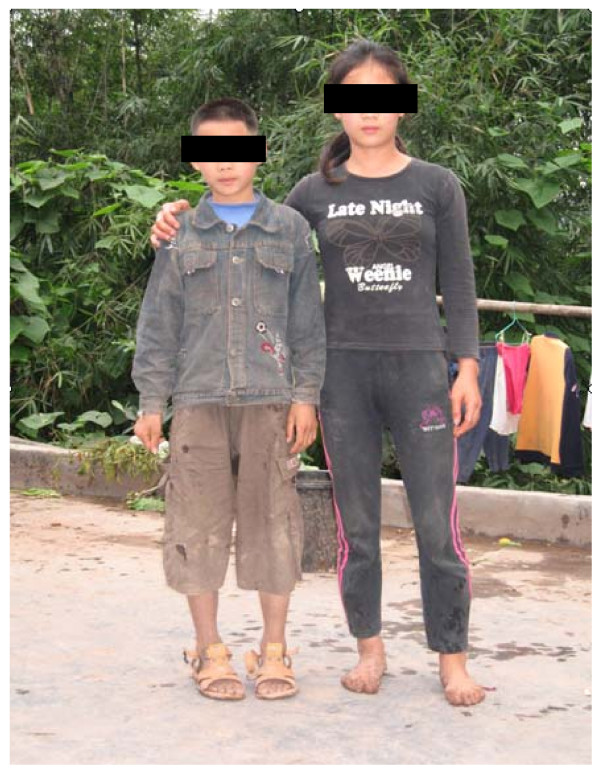
**Photo of local children without footwear in daily life (Sichuan province, China)**.

However, actual behavioral change is very slow, especially in the poorer areas. For example, local people poorly understood the link between improper behaviors and hookworm infections, such as the use of nightsoil as fertizlizer without any hygienic treatment, and consequent anemia caused by hookworm disease. Additionally, limitation of local economic development and water supplies also has a great influence on health education in rural areas. For instance, there is still no piped water for household usage in some mountain areas of Sichuan province, western China. Thus, it is difficult to make people wash their hands frequently even when they know that it is good for their health [47]. In such a scenario, health education in rural areas provides little benefit for making people understanding the linkage between hygienic behavior and improved health (Figure 3) [48].

In addition to the local efforts, the Chinese government has drawn up a national plan to improve primary health care in rural areas, with the aim of making services accessible to all rural residents, who make up about 80% of the Chinese population. It emphasizes that health education effects should become a part of the "Key Performance Indicators". According to a five-year action plan (2006-2010), the "Hundreds of Millions Farmers' Health Promotion Program" launched by the Ministry of Health, health education will be extended to 80% of rural residents in China's eastern region by 2010, and 60% in the western region, which is less developed. Furthermore, 80% of rural schools should offer health education courses. It is still likely to be a long time before the Chinese government can achieve this goal. Nevertheless, in the long term, health education is an indispensable part of the strategies to control hookworm disease [49].

## The impact of economical development on hookworm infections

**Figure 4:**
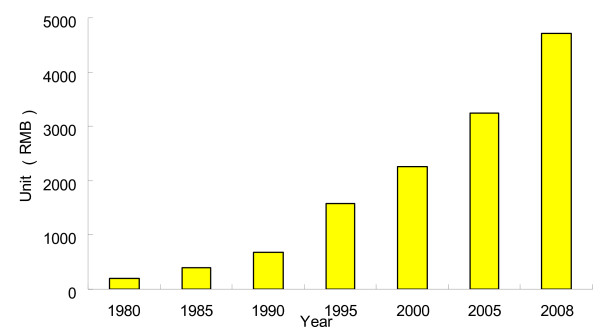
**Annual net income of farmer in China from 1980 to 2008 (April 2009 Exchange Rate: 1 US dollar = 6.85 RMB)**.

For the past thirty years, the Chinese economy has achieved a growth rate of gross domestic product (GDP) averaging nearly 10% per year. In overall size, China's economy today ranks as the fourth largest in the world in terms of total GDP at current exchange rates and in 2008 farmer's income has increased 30 times compared with 1980 (Figure 4). Nonetheless, China remains a developing nation, particularly, the average household income and consumption remain low (< 800 US $/year/person) in rural and inland areas of China. Overall, however, the strong economic performance has resulted in improved living standards for many Chinese people. About 200 million people have been brought out of poverty since the economic reforms began in 1978. By 2006, life expectancy in China reached 75 years, the infant mortality rate fell to 26 per 1,000 live births, and the literacy rate of those aged > 15 years reached to 90%. These remarkable accomplishments on accelerated urbanization, improved water and sanitation, changes of dietary pattern have made great impact on the prevalence reduction of hookworm infections in China [50].

### Accelerated urbanization development

**Figure 5:**
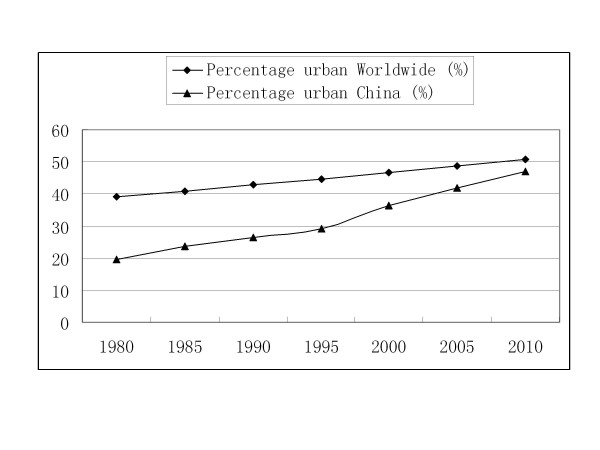
**Percentage urbanization of the world and China in 1980-2010**.

The process of urbanization has accelerated rapidly during the past 15 years in China (Figure 5). This is especially so in eastern coastal regions of China, like Zhejiang Province, where the urbanization rate was 56.6% in 2007, and in Jiangsu provinces where it was 51.9% in 2006, and in both of which high prevalence of hookworm infections was typical 20 years ago. Now, many farmers have become workers and entrepreneurs. Usually they rent their lands out and do not operate themselves in the field anymore [51]. Such land can be merged into larger agricultural fields that facilitate mechanized farming. For example, the percentage of machine cultivation, machine seeding and machine harvesting in China reached to 60%, 36%, 30% in 2008 [52], respectively, which reduced the risk for people to be exposed to soil contaminated with eggs and larvae of hookworm and other soil-transmitted helminths. In addition, mechanized farming uses no or less nightsoil as fertilizer, so there are fewer hookworm eggs developing into infective-stage larvae on the farmland.

**Figure 6:**
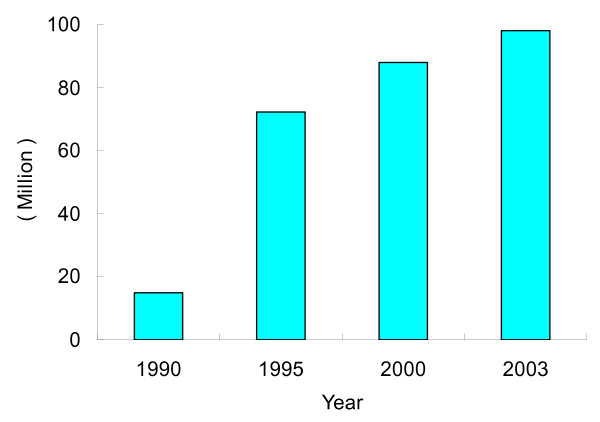
**Population of migrating workers in China from 1990 to 2003**.

However, in many areas, development and modernization continue at a slow pace [53,54]. Urbanization is short of funding and people continue to farm their land separately and, traditionally, still using human feces [55,56]. Many labourers who used to live in these areas have moved together with their families to cities attracted by high salaries for migrant workers (Figure 6). Under these much better sanitation conditions, the infection rate of hookworm has been sharply reduced among migrant workers. In urban areas, it is also easier to promote hygienic behavior by health education. In addition, medical care assurance is much better than in rural areas because of the abundant medical budget. For example, in Shanghai, the biggest city in terms of population in China, 3.65 million free person-time vaccinations were offered to migrant workers' children in 2005, additionally all migrant workers can have the same medical insurance as Shanghai citizen workers since 1 September 2002. Hence, accelerated urbanization greatly improves the living condition and health of Chinese people [57].

### Water supplies and sanitation

Access to improved water supplies and sanitation has increased significantly in China over the past two decades in parallel with economic growth. According to the official reports of the Ministry of Health, in rural areas alone, the coverage of residents with convenient access to a safe water source has increased from 49.8% in 1985 to 95% in 2006 [58].

**Figure 7:**
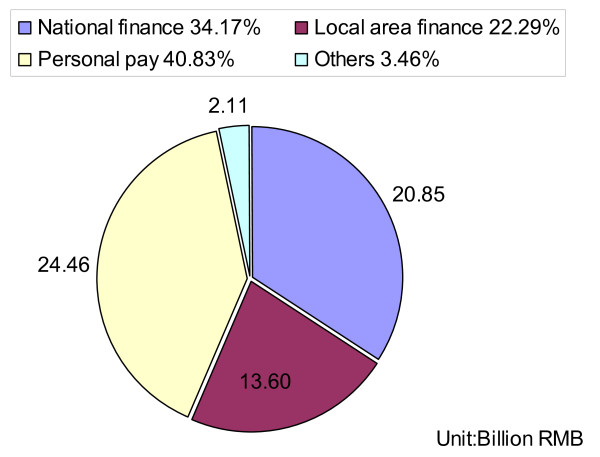
**Total investment in safe water supplies and sanitation in the Tenth Five-Year Program (2001-2005) in China**.

According to the World Bank, China differs from many other developing countries in that the end users can obtain subsidies to enable safe water ("Drinking Water Sanitary Standard", GB5749-85) supplies and sanitation, from central government and various levels of local government. In the early 2000s, there was greater emphasis on self-reliance with rural people using their own contributions and resources to improve their water supply [59]. However, in some poor rural areas, people cannot afford hygienic sanitary latrines. With the rapid economic growth, the government has added investment and increased the sum of subsidies for water supplies and sanitation. Meanwhile, the income of rural people continued to increase and, furthermore, rural people only need to pay 40% of the total costs to improve their own water supply and sanitation (Figure 7). Now, local residents are more willing to have hygienic sanitary latrines and can afford the costs (it costs about 700-800 RMB for one rural household). The improved water supply and hygienic sanitary latrines result in the reduction of hatchability of hookworm eggs and in the improvement of hygienic behavior in the rural population [60,61].

### Change of dietary pattern

Dietary patterns have changed greatly in the past 10 years in rural areas in China because of the fast economic development. Reports showed that increased consumption frequency of fats in daily diets had a positive correlation with household income [62,63]. According to the Chinese government report, the consumption of comparatively more expensive food such as fat food and animal foodstuff increased rapidly since 1992 in rural areas. Thus, rural people have a more rational dietary pattern now than they used to have. In a recent article, a model has been developed to account for the interactions between gastrointestinal parasitism and host nutrition. In order to reduce nutrition loss, the host will mount an immune response, which will affect the establishment rate of incoming larvae, mortality rate of adult worms, and fertility of female worms. This immune response depends on the health status of the body and on the food intake [64]. In animals it was shown that high-quality food maybe also directly influence the infection level of hookworm infections, but it was not shown in humans. However, improvement in diet, especially in the provision of iron, can improve homeostasis and regulation of red blood cell supply [65]. Thus, change of dietary pattern of Chinese rural people helped to promote recovery after deworming. For example, it improves anemia which was caused by hookworm disease and reduces the opportunity of stunting among children who are infected with hookworm [66-68].

Various infection modes have been noticed among different age groups. For example, adults become infected with hookworm mostly by working in the untreated nightsoil contaminated farmland, whereas children become infected with hookworm mostly by playing in contaminated fields without wearing shoes or play with the contaminated soil by hand. These age-related patterns of behavior add yet another level of complexity for those attempting to control infections in the long-term (Table 3).

**Table 3:** Comparison of dietary patterns between urban area and rural area in China in 1992 and 2002 [68]

**Dietary patterns**	**Urban area**		**Rural area**	
	
	**1992**	**2002**	**1992**	**2002**
Grain Foodstuff (%)	57.4	47.4	71.7	60.7
Animal Foodstuff (%)	15.2	19.2	6.2	11.6
Fat Food (%)	28.4	35.4	18.6	27.7

## Discussion

Hookworm infection remains a worldwide public-health problem as long as poverty persists in the developing world. In Japan, it has been proved that poverty reduction, urbanization and large-scale control programs were the most effective means for reducing the prevalence and intensity of hookworm infections [54]. Economic prosperity and the reduction of agriculture populations have also contributed greatly to the control of hookworm and other diseases of poverty including tuberculosis and malaria. Now the Chinese economy increases even faster than that of Japan during the 1960s-1970s. Therefore, it is reasonable to predict that steady economic improvements and urbanization in China will also lead to great reductions in hookworm transmission.

Two methods for direct diagnosis, e.g. Baermann method and Koga agar plate, are widely used in many research institutions to detect human helminth infections which including hookworm infections [69,70]. But in most of the local CDCs in China, people still use traditional Kato-Katz method to detect hookworm infections. So it is important for the CDCs in China to provide more training courses on helminths diagnosis. In addition, the free vaccine list used in residents has been expanded 3 times more than that number in the past 5 years in China. So it is possible that all the people under the threat of hookworm infections will be able to take free vaccine in the future, if vaccine to prevent from hookworm infections is available for human use, which will be a good way to control hookworm transmission [71]. Furthermore, tribendimidine has been approved by the Chinese State Food and Drug Administration in 2004 as an anthelmintic drug which is effective against hookworm [72]. Some data show that the cure rate of tribendimidine against hookworm infection was 82.0% and its anti-parasite spectrum includes 14 species [73,74]. Thus it is suggested to try this medicine for large-scale deworming in the near future.

Owing to the excellent work of the Chinese government and the economic prosperity of China, the prevalence and intensity of hookworm infections is likely to decline sharply in the next decade. Nonetheless, the gap between the rich and the poor is still widening, and the inequity of economic and social development among different regions of China is going to exist for a long period. Consequently it is a laborous task to eliminate hookworm in the poorer regions in China. The regions under worst threat of hookworm infections are mainly located in the southern and central China, where the soil-transmitted helminth infection rates are still as high as 20.1%~56.2% [75]. In these regions, it is necessary to put more investment into prophylaxis and treatment of hookworm infections. Therefore, the three-pronged approach, e.g. distributing anthelmintic drugs in schools and undertaking large-scale deworming of hookworms, improving water supplies and sanitation, and proper health education on good-living habits, will still be the most effective strategy for controlling hookworm infections in China. With this approach, it is believed that hookworm transmission could be well-controlled or even interrupted in the near future.

## Competing interests

The authors declare that they have no competing interests.

## Authors' contributions

QZ, YC, XNZ conceived the study, carried out data collection and analysis and drafted the manuscript. HBZ, JXC, XNZ conceived the project, assisted in the interpretation of the results and revised the manuscript. All authors read and approved the final manuscript.
